# The Concept of Health Debt Incurred during the COVID-19 Pandemic on the Example of Basal Cell Skin Cancer Diagnosis in Lower Silesia

**DOI:** 10.3390/jcm13164923

**Published:** 2024-08-21

**Authors:** Danuta Szkudlarek, Tomasz Gębarowski, Nikola Hauzer, Benita Wiatrak

**Affiliations:** 1 Pathology Department, Provincial Hospital Center of the Jelenia Góra Valley, Ogińskiego 6, 58-506 Jelenia Góra, Poland; 2 Department of Biostructure and Animal Physiology, Wrocław University of Environmental and Life Sciences, Kożuchowska 1, 51-631 Wrocław, Poland; tomasz.gebarowski@upwr.edu.pl; 3 Veterinary Biotechnology Student Science Club “Refectio”, Department of Biostructure and Animal Physiology, Faculty of Veterinary Medicine, Wrocław University of Environmental and Life Sciences, Kożuchowska 1, 51-631 Wrocław, Poland; 4 Department of Pharmacology, Faculty of Medicine, Wrocław Medical University, Mikulicza-Radeckiego 2, 50-345 Wrocław, Poland

**Keywords:** COVID-19 pandemic, BCC, healthcare

## Abstract

**Introduction:** This study explores the impact of the COVID-19 pandemic on the diagnosis of basal cell carcinoma (BCC) in Lower Silesia, Poland, comparing pre-pandemic, pandemic, and post-pandemic periods. It investigates how different medical facilities adapted to the pandemic’s challenges and the subsequent implications for cancer diagnosis. **Methods:** Data from histopathology and cytology laboratories were analyzed, focusing on BCC diagnoses from 2018 to 2022. This study included various medical centers categorized by size and source of implementation. Statistical analyses were conducted to compare diagnoses before, during, and after the pandemic. **Results:** During the initial wave of the pandemic, there was a significant reduction in newly diagnosed BCC cases, followed by a surge post-pandemic. Larger medical centers adapted more effectively, while district hospitals faced challenges. Private practices maintained stable diagnosis rates. The increase in diagnoses post-pandemic suggests a backlog of undiagnosed cases during the pandemic. **Discussion:** Challenges in accessing healthcare during the pandemic led to delayed cancer diagnoses. Larger medical centers were better equipped to handle the crisis, while district hospitals struggled. Private practices maintained stability, possibly due to pre-scheduled appointments. Recommendations include public education on symptom recognition and standardizing histopathological evaluation protocols. **Conclusions:** Despite data limitations, this study provides valuable insights into the pandemic’s impact on cancer diagnosis, highlighting the need for proactive measures in future health crises to ensure timely detection and treatment of cancer cases.

## 1. Introduction

On 11 March 2020, the World Health Organization (WHO) declared a pandemic due to the SARS-CoV-2 virus, which causes COVID-19. This declaration came in response to the virus’s global spread and the significant number of illnesses and deaths that followed. The pandemic status of COVID-19 highlighted the urgent need for a coordinated international response to this health crisis [1].

The first case of COVID-19 in Poland was confirmed on 4 March 2020. Subsequently, the infection rate began to escalate, leading to the implementation of initial restrictions by Prime Minister Mateusz Morawiecki on 12 March 2020. These measures included school closures, bans on gatherings, border shutdowns, limitations on public transportation, and restrictions on various commercial activities. In Lower Silesia and throughout Poland, specific actions were taken according to the evolving epidemic situation, which ranged from business operation limits, public event constraints, trade restrictions during certain hours, to modifications in educational settings [2,3].

The COVID-19 pandemic has had a profound impact on society and healthcare systems globally, posing numerous challenges and consequences [4,5]. Its effects have been wide-ranging, with lockdowns, quarantines, and social distancing measures significantly disrupting the economy, education, social relationships, and everyday life. As a result, unemployment rates increased, businesses encountered economic hardships, and people were forced to adjust to new societal norms.

The healthcare sector’s response to the pandemic underscored the critical need for adaptability, resource allocation, and accessibility. Healthcare systems worldwide were strained, contending with a shortage of hospital beds, medical equipment, and healthcare personnel [6,7]. The shift towards telemedicine and remote healthcare services became imperative, along with the adoption of effective infection control practices. Previously used telemedicine solutions were continued, e.g., in rehabilitation, and new systems were also developed, e.g., in dermatology [8,9]. One significant consequence of the pandemic is the emergence of what has been termed “health debt.” Health debt refers to the backlog of untreated or undiagnosed health conditions that accumulate during a crisis like a pandemic when healthcare resources are diverted to urgent care and routine medical services are postponed or reduced. This debt can lead to delayed diagnoses and treatments, worsening health outcomes for patients with conditions unrelated to the pandemic [6].

The pandemic’s restrictions, including lockdowns and limited healthcare access, led to significant delays in diagnosing and treating critical health conditions such as cancer, cardiovascular diseases, and neurodegenerative diseases [10]. Fear of infection and challenges in accessing medical facilities caused many patients to delay seeking medical attention, which in turn delayed diagnoses and treatments. The focus on COVID-19 patients also meant that routine medical tests, monitoring, and procedures for managing chronic diseases were often neglected. Furthermore, the pandemic disrupted clinical and scientific research, potentially causing setbacks in the development of new medical therapies and drugs, as well as hindering access to innovative treatments [1,10].

The pandemic’s strain on healthcare systems led to challenges in providing immediate care for non-COVID-19 conditions, potentially compromising treatment quality. The associated stress may have exacerbated mental health issues among patients and healthcare workers, impacting the overall healthcare delivery [11,12]. Despite the pandemic’s peak between March 2020 and May 2022, circulatory system diseases remained the leading cause of death in Poland and worldwide, with ischemic heart disease, stroke, and peripheral vascular diseases being the most prevalent [13]. Malignant cancers were the second leading cause of death [13,14].

The incidence of various types of skin cancer varies based on geographic region, lifestyle, genetic factors, and other variables [15,16]. Skin cancers, which are characterized by low mortality but are the most frequently diagnosed, are divided into three main types: melanomas, non-melanocytic skin cancers, and other skin cancers [17]. The three primary types are as follows: basal cell carcinoma (BCC), the most common, develops on sun-exposed skin and has a low risk of distant metastasis [15,18]; squamous cell carcinoma (SCC), associated with long-term sun exposure, is slightly more malignant than BCC and capable of metastasizing [15]; Merkel cell carcinoma, a rare but aggressive form, develops from tactile stimulus-receiving Merkel cells [19]; melanomas, which develop from melanocytes, are rarer but more malignant and have the potential to metastasize. BCC and SCC are the most frequently diagnosed, accounting for the majority of skin cancer cases [15,16,18].

Skin cancer, particularly BCC and SCC, typically exhibits a very low mortality rate. Both forms of cancer have a benign clinical nature, seldom metastasize, and can often be effectively treated, especially when diagnosed early. Regular monitoring of skin lesions, using sun protection, and being aware of symptoms are crucial for early diagnosis and treatment [16]. The cure rate for BCC is typically very high, especially when detected and treated promptly. The incidence and cure rate of BCC may vary based on various factors, including early detection, lesion location, and BCC subtype (there are several subtypes, each with slightly different properties and prognoses) [20].

Overall, the mortality rate from skin cancer remains relatively low when compared to other forms of cancer. Nevertheless, it is imperative to implement preventive measures to mitigate the risk of skin cancer and enhance overall health outcomes. Such measures include regular examination of skin lesions, diligent use of sun protection, and minimizing excessive UV radiation exposure. Should there be any concerns or noticeable changes in the skin, seeking a comprehensive evaluation and potential treatment from a dermatologist is highly recommended. Regrettably, obtaining precise statistics on the global and Polish incidence and cure rates for basal cell carcinoma (BCC) can be challenging due to discrepancies in reporting practices and the tendency for BCC to be treated within dermatological settings rather than oncological ones [21,22]. These data are often gathered by a variety of entities, including public health organizations and research institutes. Regardless, the importance of early monitoring of skin alterations and immediate consultation with a healthcare provider upon observing any worrisome changes cannot be overstated. The prevention and early detection of skin cancers, BCC included, are crucial for efficacious treatment and favorable prognoses [23,24,25].

The concept of health debt is particularly relevant in the context of cancer diagnosis and treatment, where timely detection is crucial for effective management and prognosis. Numerous studies from various countries have reported significant reductions in cancer screenings and diagnoses during the pandemic, leading to concerns about the long-term impact on cancer-related morbidity and mortality. For instance, studies from the United Kingdom, the United States, and Italy have documented delays in the diagnosis of breast cancer, colorectal cancer, and other malignancies due to pandemic-related healthcare disruptions. These delays have raised fears of increased cancer stages at diagnosis and subsequent treatment challenges, contributing to a growing health debt [26,27,28,29].

This study explores the concept of health debt incurred during the COVID-19 pandemic, focusing on its impact on the diagnosis of basal cell carcinoma (BCC) in Lower Silesia, Poland. By comparing the pre-pandemic, pandemic, and post-pandemic periods, we aim to evaluate the impact of the pandemic on diagnostic processes and their availability during and after the peak periods of COVID-19.

## 2. Materials and Methods

### 2.1. Database

This study utilized data obtained from histopathology and cytology laboratories within pathology departments, which assessed specimens from hospitals and clinics representing a cross-section of provincial hospitals, district hospitals, university hospitals, and private medical practices in Lower Silesia. The analysis focused on the number of positive basal cell carcinoma (BCC) test results assessed monthly from 2018 to 2022. The study findings were categorized based on the size of the medical centers, with a differentiation between small and large centers determined by the population of the respective cities. Large centers encompass hospitals and clinics in Wrocław (population > 500,000), while other district hospitals in Lower Silesia were deemed too small for inclusion in this analysis. It is important to highlight that, owing to their distance from the provincial capital, these hospitals serve as the primary medical facilities for residents of the districts.

The data were analyzed based on the number of tests performed during and outside the pandemic, as well as before and after the pandemic. Furthermore, the impact of the first wave of the pandemic (covering the 4 months from March to June 2020) on the number of diagnosed cases was assessed in comparison to the later stages of the epidemic, as well as before and after the epidemic in Poland.

Furthermore, the data division considered the source of implementation, distinguishing between results originating from hospitals, smaller medical centers, and private practices. The statistical analysis exclusively incorporated results coded according to the International Classification of Diseases, version 10 (ICD-10).

This study included 5 large medical centers, 8 smaller centers, and 10 private practices.

### 2.2. Study of Population and Variables

This study encompassed all histopathological test results, irrespective of age, from the Lower Silesia region. Data from the Lower Silesian Oncological Center were excluded due to the hospital’s closure during the first wave of the pandemic. The included dataset spans from January 2018 to December 2022 (n = 2992). Only patients with no prior history of cancer diagnosis were considered in this study. Cancer diagnoses were categorized based on the location: skin (ICD-10: C43 and C44; dermatology and general practice).

### 2.3. Statistical Analyses

Statistical analyses were conducted using Statistica 14.1 software (TIBCO Software Inc., Palo Alto, CA, USA). A *p*-value of <0.05 was deemed statistically significant. Differences between medical centers were assessed using Friedman’s ANOVA with appropriate post-hoc tests. Wilcoxon tests were employed to compare the number of new BCC diagnoses per practice each month across the individual analyzed periods: pre-pandemic, first wave of the pandemic, epidemic period, and post-pandemic.

## 3. Results

### 3.1. Patients’ and Practices’ Characteristics

To investigate the potential impact of the COVID-19 pandemic on BCC diagnostics in Lower Silesia, Poland, this study compared the number of new cancer diagnoses in eight district hospitals, four hospitals in Wrocław, and private clinics during the pre-pandemic period from January 2018, throughout the epidemic in Poland (March 2020–May 2022), and during the initial months following the pandemic until 31 December 2022 (for detailed information, see Section 2). The number of diagnosed cases in individual types of medical facilities is summarized in Table 1. A total of 3207 patients diagnosed with cancer in the analyzed time were included in this study. Interestingly, the diagnosis of BCC was more often made in small centers than in large ones. Before the pandemic (January 2018–February 2020), large facilities diagnosed an average of 7.54 cases monthly, small facilities had 43.96 cases, and private practices saw an average of 3.12 cases. During the initial wave of the pandemic from March to June 2020, district hospitals reported 17 cases of BCC per month, large hospitals in Wrocław identified an average of 4.75 cases, and 2 cases per month were diagnosed in private practices. Throughout the epidemic in Poland (March 2020–May 2022), the numbers fluctuated, with 32.74 cases per month in smaller facilities, 7.89 in large facilities, and 3.52 in private practices. Following the pandemic, there was a significant increase in diagnosed cases across all three types of medical centers: district hospitals saw 64 cases monthly, large hospitals in Wrocław recorded an average of 14.43 cases, and private practices reported 6.57 cases per month.

### 3.2. Reduced Number of New BCC Diagnoses during the First Wave of the COVID-19 Pandemic

At the outset of our investigation, we posited that the COVID-19 pandemic might adversely affect BCC diagnostics in Lower Silesia, Poland, with the impact varying based on the size of the medical facility. Hence, we compared the average number of new diagnoses per cancer diagnosis between 2018–2019 and the same period in 2020, specifically from March to June.

**Figure 1:**
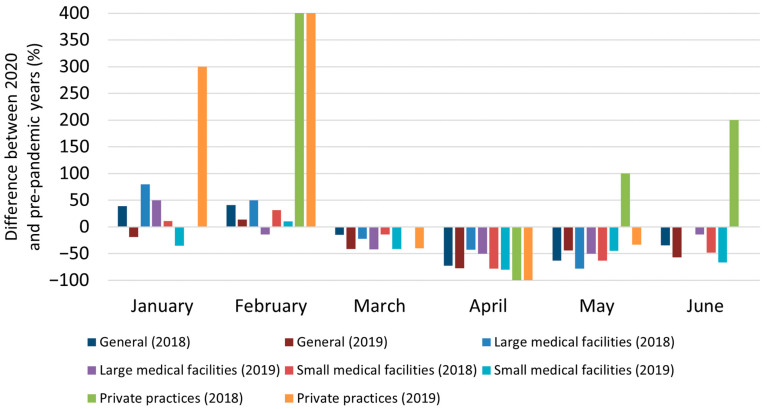
Differences in new BCC diagnoses in Lower Silesia, Poland, in the early months of the pandemic compared to previous years depending on the type of medical facility.

Interestingly, while the number of new BCC diagnoses per general practice remained comparable between January to February 2019 and January to February 2020, a notable decrease in new cancer diagnoses per medical clinic was observed during the March–June 2020 period compared to the corresponding months in 2018 (March: −14.9%, April: −72.5%, May: −62.7%, June: −34.2%) and 2019 (March: −41.2%, April: −77.6%, May: −44.1%, June: −56.9%). This trend was consistent across different sizes of medical centers, including private practices (Figure 1).

Indeed, the number of new BCC diagnoses in large centers during the first wave of the pandemic decreased by 38.7% (*p* < 0.05) compared to the same period in 2018 and 2019. The number of diagnoses in district hospitals showed a comparable decrease (2018: −51.1%, 2019: −57.8%, both *p* < 0.05). Finally, in private practice, a reduction in newly diagnosed BCC cases was only observed when comparing to 2019 (−52.9%, NS).

### 3.3. Reduced Number of New BCC Diagnoses during the COVID-19 Epidemic

**Figure 2:**
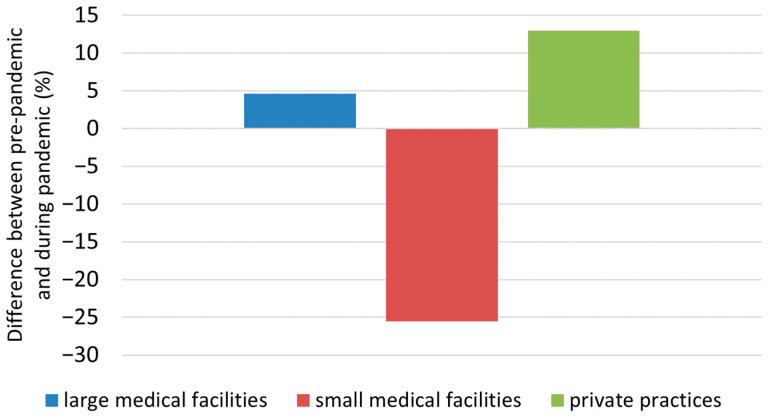
Differences in the number of new BCC diagnoses in Lower Silesia, Poland, before the pandemic and during the epidemic by medical center.

Another hypothesis considered was whether larger medical centers could adapt more swiftly to the pandemic and resume diagnostics and treatment for other diseases compared to patients infected with the SARS-CoV-2 virus. To explore this, we compared the average number of diagnosed BCC cases per month from 26 months before the pandemic and during the epidemic in Poland (March 2020–May 2022). The differences are shown in Figure 2.

In the case of district hospitals, a consistent decrease in the percentage of BCC diagnoses was observed throughout the entire epidemic period (−25.5%; *p* < 0.05). Notably, there was no statistically significant difference in BCC diagnosis rates in hospitals located in the capital of Lower Silesia. Interestingly, in private practices during the entire epidemic period in Poland, a higher number of BCC diagnoses was observed (by 12.9%; NS) compared to the period preceding the pandemic.

### 3.4. Increased BCC Diagnostics after the COVID-19 Pandemic

**Figure 3:**
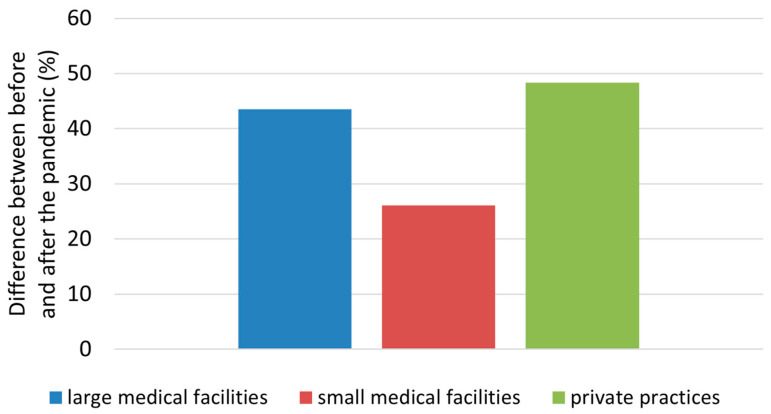
Differences in the number of new BCC diagnoses in Lower Silesia, Poland, before and after the pandemic by medical center.

The third hypothesis posited an escalation in the incidence of BCC during the post-epidemic period, irrespective of the medical center. As anticipated, all three types of analyzed medical centers exhibited a statistically significant surge of approximately 40% in diagnosed BCC cases, potentially attributed to the phenomenon known as “health debt”. The increase in diagnosed cases varied by medical center, with the smallest increase at 26.1% noted in district hospitals, a 43.6% increase in large medical facilities, and the largest increase, approximately 48.4%, occurring in private practices (Figure 3).

### 3.5. Comparison of Macroscopic Findings with Histopathological Diagnosis

While histopathological evaluation remains the gold standard, dermatoscopy has been shown to improve the accuracy of clinical diagnosis of BCC, as recommended by the “European consensus-based interdisciplinary guideline for diagnosis and treatment of basal cell carcinoma—update 2023” with a grade recommendation A and a strength of consensus of 100% [30]. Dermatologists and plastic surgeons often rely on dermatoscopy to assess nevi, particularly those exhibiting benign characteristics. The importance of teledermatology, including the evaluation of clinical and dermoscopic images, should also be emphasized. Teledermatology, particularly teledermoscopy, has emerged as a valuable tool in the early detection and monitoring of atypical melanocytic lesions. De Giorgi et al. have discussed the challenges and limitations of short-term teledermoscopic monitoring, emphasizing the need for careful patient selection and follow-up to avoid missing potential melanomas, particularly those that may not initially exhibit clinical or dermoscopic atypia [31]. In addition, Tognetti et al. explored the efficacy of using various electronic devices for teledermoscopic diagnosis [32]. Their study found that even with the use of small-screen mobile devices, there was no significant reduction in diagnostic accuracy compared to traditional large-screen devices, underscoring the feasibility of teledermatology in diverse settings. These findings support the integration of teledermoscopy in routine clinical practice.

**Figure 4:**
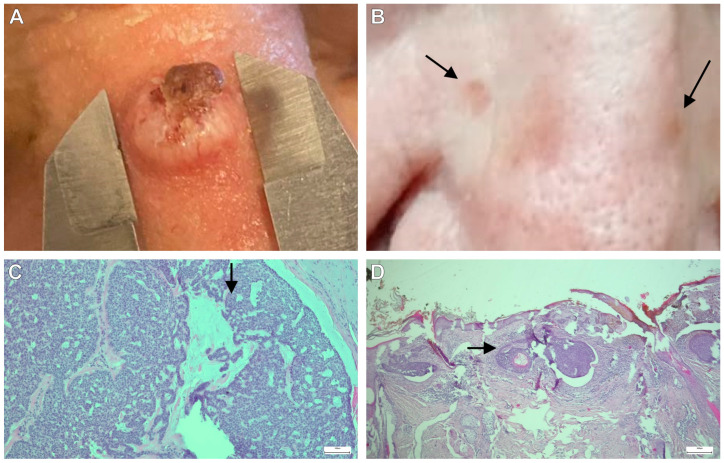
Examples of diagnoses of two clinical cases: (**A**,**B**) lumps on the nose; (**C**) histopathological diagnosis of BCC; (**D**) histopathological diagnosis of trichoepithelioma.

In Figure 4, we present two illustrative cases: (A) a nasal skin tumor with surface ulceration and (B) a skin tumor on the nose. Both initially received benign assessments based on clinical observations. However, subsequent histopathological analyses revealed that the former was diagnosed as BCC. Figure 4C exhibits basaloid lesions encased by a palisade layer, with potential epidermal ulceration and a stroma displaying myxoid/desmoplastic/amyloid/collagen characteristics. Conversely, the latter case was diagnosed as trichoepithelioma—a small nodular lesion appearing skin-colored macroscopically. Yet, as depicted in Figure 4D, small spherical basaloid foci resembling pilosebaceous–apocrine buds were observed, devoid of any atypia or pathological mitoses.

## 4. Discussion

Between March and June 2020, a significant decrease in the incidence of newly diagnosed skin cancer cases was observed in Lower Silesia compared to both pre-pandemic levels and the same period in 2019. This reduction was uniform across medical facilities of all sizes, including smaller ones, throughout the entire epidemic duration in Poland. Larger medical facilities showed a faster response to the pandemic, implementing new restrictions more promptly, which may indicate that the residents of Wrocław were less reluctant to seek medical care compared to those in smaller towns and rural areas.

Notably, after the epidemic, there was a marked increase in new cancer diagnoses across all groups, exceeding the numbers seen during both the pre-pandemic and pandemic periods. This significant finding suggests a backlog of undiagnosed cases accumulated during the pandemic. To our knowledge, this study is the first to examine the impact of the COVID-19 pandemic and the ensuing lockdown on skin cancer diagnoses in Lower Silesia, Poland, with a particular emphasis on the size of the medical facility, covering the time frames before, during, and after the pandemic.

The findings of this study are consistent with the existing literature, which has performed similar analyses in various countries, including the Netherlands, the United States, Great Britain, and Germany. Notably, in the Netherlands, a significant reduction in new cancer diagnoses, ranging from 26 to 60%, was observed during a week of restrictions (6 to 12 April 2020) [33]. In the United States, there was a weekly decrease of 46.4% in new diagnoses [34]. The United Kingdom also saw a considerable reduction, with new diagnoses falling by 58% [35]. In Germany, a decrease of 42.9% in skin cancer cases was noted in April 2020 [36]. Other studies have indicated similar trends in various countries.

Our research, along with studies from other nations, allows us to propose two hypotheses to explain the decline in new cancer diagnoses, especially during the initial phase of the COVID-19 pandemic. Patients exhibiting symptoms of skin lesions faced difficulties in obtaining medical care. Moreover, patients with skin cancer, due to its typically mild clinical progression, could afford to be diagnosed later without the risk of death from advanced cancer [17,37]. The pressure on hospital capacities caused by COVID-19 infections led to fully occupied beds, the erection of temporary hospitals for virus-infected individuals, and limitations on doctor availability. Further compounding these issues were additional measures such as shortened working hours for medical staff, quarantines for healthcare workers, and restricted access to Emergency Departments due to visitor restrictions [38,39,40].

Wrocław, the capital of Lower Silesia, is home to a high concentration of specialists and state-of-the-art medical infrastructure. However, access to healthcare can be limited in smaller towns and rural areas. The distribution of district hospitals and specialized healthcare institutions is significantly less dense outside of Wrocław, resulting in longer travel distances for residents seeking medical services. Furthermore, the elderly and those without private transportation face challenges due to the limited availability of public transport, which impedes their ability to access hospitals. During the peak of the pandemic, the surge in hospital admissions and the strain on available beds and intensive care units, coupled with high infection rates, placed a substantial burden on local healthcare systems. Additionally, various restrictions, including lockdowns and operational limitations on medical facilities, impacted the accessibility and effectiveness of healthcare services [41,42].

Interestingly, the Netherlands saw a decrease in Emergency Department usage even before formal restrictions on hospital access were implemented [33]. This suggests that a potential fear of infection may have led to a reduction in diagnosis and treatment for both chronic and cancerous conditions [43].

As underscored in our study, larger medical centers in Lower Silesia were more effective in adapting to the conditions of the epidemic. They managed this by segregating COVID zones and maintaining routine operations in specialist clinics throughout the entire epidemic period in Poland. This is in stark contrast to district hospitals, which, due to their smaller infrastructures and fewer medical staff, found it challenging to adapt quickly. Many district hospitals were converted into COVID facilities, and the need to travel to the capital of Lower Silesia deterred patients from seeking specialized care locally [44].

In the United States, patients openly voiced their concerns about hospitals serving as sources of infection. The fear of contracting the virus during the pandemic led to a hesitancy to visit medical clinics, even when experiencing symptoms such as skin abnormalities, breast lumps, or abdominal pain [34]. A similar reluctance was observed in Italy, with patients suspected of having breast cancer refusing surgery due to fears of contracting COVID-19 [45].

The initial surge in COVID-19 cases during the first wave of the pandemic overwhelmed healthcare systems, resulting in delayed or suspended services in public facilities [46]. Those who could afford it turned to private healthcare options in response to concerns about their health. Despite the pandemic, there was no statistically significant decrease in the number of newly diagnosed skin cancers in private laboratories, regardless of the period of the epidemic analyzed. This observation may be attributed to the nature of private visits, where patients are scheduled for specific times, facilitating the implementation of social contact restrictions and adherence to rigorous disinfection procedures between consecutive patients.

Furthermore, in the public health sector, teleconsultations became the preferred method post-outbreak for triaging and postponing less urgent cases, particularly those related to chronic diseases [47,48]. Skin lesions, including cases of basal cell carcinoma (BCC), the most frequently diagnosed cancer with a low mortality risk, were deemed non-urgent. The focus on prioritizing urgent cases likely contributed to the relatively stable rate of skin cancer diagnoses in private clinics.

An intriguing finding from a survey involving nearly 1000 dermatologists in the United States revealed that the number of patients and biopsies conducted in one week in March 2020 was less than double compared to a week in February 2020 [49]. This surge in activity underscores the efforts made by dermatologists to promptly address concerns, possibly facilitated by the adaptability of private practices to implement necessary precautions during the pandemic.

Interestingly, the diagnosis of basal cell carcinoma (BCC) is more frequently made in smaller centers than in larger ones, likely due to its low mortality risk. It is noteworthy that, regardless of the size and nature (public/private) of the medical facility, a statistically significant increase in the diagnosis of BCC was observed in Lower Silesia following the epidemic period. This phenomenon could be characterized as a “repayment of health debt”. Patients who deferred diagnostic tests during the epidemic returned in the second half of 2022, following the relaxation of restrictions. This sudden rise in BCC diagnoses, even when compared to the years 2018–2019, underscores the nature of this cancer with its relatively low-risk metastatic potential and almost negligible mortality.

In contrast, for other cancers, delayed diagnoses during the pandemic may lead to increased mortality due to ineffective therapy. To address this, a proactive approach of “prevention rather than cure” is crucial in preparing for any future pandemics. This involves public education to enhance awareness that the risk of infection is relatively low when following effective protective measures. Furthermore, the public should be informed about recognizing symptoms that warrant immediate consultation in the case of skin cancer and the significance of the acronym ABCDE in the case of melanomas [50].

The primary strengths of this study lie in the comprehensive inclusion of medical facilities in the analyses and the use of representative data that cover both public and private centers in Lower Silesia. Importantly, the evaluation of newly diagnosed cases both before and after the official conclusion of the epidemic in Poland, along with the thorough consideration of the entire epidemic period, including its initial waves, significantly enhances the value of this research.

However, it is essential to acknowledge certain limitations within this study. The diagnosis of cancer was solely based on histopathological results using ICD-10 codes, without access to critical clinical information such as variations in the timing of lesion occurrence, the timeline of the first medical consultation, and the diagnostic process. Furthermore, the data from private practices may be potentially inaccurate, as not all removed skin lesions undergo histopathological evaluation by dermatologists and plastic surgeons.

The removal of skin changes can be performed either with or without a histopathological evaluation, with the final decision depending on several factors, including the characteristics of the skin change, suspicion of malignancy, and the dermatologist’s judgment. Moles that exhibit concerning features such as irregular edges, uneven pigmentation, changes in shape, itching, bleeding, or rapid growth may prompt the doctor to conduct a biopsy for histopathological assessment. Microscopic analysis aids in determining the risk of malignancy, which is crucial for cases suspected of melanoma or other malignant skin tumors. Early detection and treatment are vital for improving prognosis, underscoring the importance of accurate diagnosis.

Even in the case of benign lesions, some doctors may choose to conduct a histopathological analysis to ensure safety [51]. In instances where malignancy is suspected, particularly with melanoma, a histopathological examination is always recommended for an accurate diagnosis. While errors related to the absence of a histopathological assessment are rare, they can occur. Therefore, there is a general recommendation to perform a histopathological analysis for any concerning or suspicious skin lesions. These limitations underscore the importance of interpreting the findings with caution and recognizing the need for complementary research approaches in future studies.

In our view, dermatologists frequently use a dermatoscope for mole assessment, evaluating skin structure, features related to melanoma, and clinical characteristics. Dermatoscopy is non-invasive and effective for early lesion diagnosis, although it may pose challenges for less experienced practitioners. Histopathology is considered the gold standard for skin cancer diagnosis, providing confirmation or exclusion of cancer cells, identifying the type of cancer, assessing malignancy, and offering a prognosis [51,52,53,54,55]. To ensure a standard protocol, it is suggested that legal regulations be established for the histopathological evaluation of all removed lesions. This could prevent errors among less experienced dermatologists. In private practice, where an additional fee might be required for a consultation with a pathology specialist, histopathological examinations may be omitted.

While basal cell carcinoma (BCC) typically exhibits slow, local growth and a low degree of malignancy, distant metastases are rare. It is predominantly associated with UV radiation exposure, and surgical removal is the primary treatment, with recurrences being rare. Despite its characteristics, BCC requires attention and treatment. Any non-healing, bleeding, or changing skin lesion should be brought to the attention of a doctor. Regular skin examinations and minimizing sun exposure are essential for preventing this type of skin cancer.

## 5. Conclusions

During the COVID-19 pandemic, numerous restrictions were observed in both social life and access to healthcare. A prime example of the challenges in oncological diagnosis is the number of diagnosed cases of BCC. Due to the benign and prolonged course of this cancer, significant difficulties exist in accessing public healthcare in Lower Silesia. The simultaneous increase in diagnosed cases after the lifting of restrictions perfectly fits the definition of the concept of health debt.

Patients with symptoms of skin changes encountered difficulties in obtaining medical care. Moreover, patients with skin cancer, due to its typically mild clinical progression, could afford to be diagnosed later without the risk of death from advanced cancer. The pressure on hospital capacities caused by COVID-19 infections led to fully occupied beds, the erection of temporary hospitals for virus-infected individuals, and limitations on doctor availability. Further compounding these issues were additional measures such as shortened working hours for medical staff, quarantines for healthcare workers, and restricted access to Emergency Departments due to visitor restrictions.

In conclusion, the COVID-19 pandemic had a significant impact on the diagnosis and treatment of cancer worldwide. Fear of infection and restrictions on healthcare access led to a significant reduction in the number of new cancer diagnoses. In the case of skin cancer, despite its relatively low risk, delays in diagnosis and treatment can lead to serious health consequences. Therefore, it is important for patients to be aware that the risk of infection is relatively low when following effective protective measures, and any non-healing, bleeding, or changing skin lesion should be consulted with a doctor. Regular skin examinations and minimizing sun exposure are key to preventing this type of skin cancer. Finally, it is important for future research to continue analyzing the impact of the pandemic on cancer diagnosis and treatment to better prepare for future pandemics.

## Figures and Tables

**Table 1:** Average number of diagnosed cases per month in various types of medical facilities.

	Large Medical Facilities	Small Medical Facilities	Private Practices
Before the pandemic (January 2018–February 2020)	7.54	43.96	3.12
During the epidemic (March 2020–May 2022)	7.89	32.74	3.52
The first wave of the pandemic (March–June 2020)	4.75	17.00	2.00
After the pandemic (June–December 2022)	14.43	64.00	6.57

## Data Availability

Data is contained within the Supplementary Materials.

## Supplementary Materials

The following supporting information can be downloaded at: https://www.mdpi.com/article/10.3390/jcm13164923/s1.
